# Cold Plasma Irradiation Attenuates Atopic Dermatitis *via* Enhancing HIF-1α-Induced MANF Transcription Expression

**DOI:** 10.3389/fimmu.2022.941219

**Published:** 2022-07-14

**Authors:** Tao Sun, Xinru Zhang, Chao Hou, Shujun Yu, Yujing Zhang, Zhuo Yu, Ling Kong, Changqing Liu, Lijie Feng, Dong Wang, Guohua Ni

**Affiliations:** ^1^ Hefei Institutes of Physical Science, Chinese Academy of Sciences, Hefei, China; ^2^ University of Science and Technology of China, Hefei, China; ^3^ School of Basic Medical Sciences, Anhui Medical University, Hefei, China; ^4^ Department of Obstetrics and Gynecology, The First Affiliated Hospital of Anhui Medical University, Hefei, China

**Keywords:** cold atmospheric plasma, mesencephalic astrocyte-derived neurotrophic factor, hypoxia-inducible factor-1α, nuclear factor kappa-B, atopic dermatitis

## Abstract

Cold atmospheric plasma has been widely applied in medical treatment clinically, especially skin diseases. However, the mechanism of cold atmospheric plasma on the treatment of skin diseases is still undefined. In this study, dinitrofluorobenzene-induced atopic dermatitis mice model was constructed. Cold atmospheric plasma was able to decrease skin cells apoptosis, relieve skin inflammation, ER stress and oxidative stress caused by dinitrofluorobenzene stimulation, which was mediated by cold atmospheric plasma-induced MANF expression. In terms of mechanism, hypoxia-inducible factor-1α expression was increased intracellularly after cold atmospheric plasma treatment, which further bound to the promoter region of *manf* gene and enhanced MANF transcriptional expression. This study reveals that cold atmospheric plasma has a positive effect on atopic dermatitis treatment, also demonstrates the regulatory mechanism of cold atmospheric plasma on MANF expression *via* HIF-1α, which indicates the potential medical application of cold atmospheric plasma for atopic dermatitis treatment.

## Introduction

Atopic dermatitis (AD) is a chronic disease of skin with characteristics of relapse and skin inflammation, which has a rising incidence worldwide (1). AD’s pathogenesis is very complex to be barely defined currently. Multiple factors have been reported to be closely associated with the occurrence and development of AD, including immune dysfunction, skin function failure and environmental changes (2). It has been proven that Endoplasmic Reticulum Stress (ER stress) and oxidative stress responses play a pivotal pathogenic role for AD (3, 4). At present, AD’s therapy is mainly based on corticosteroid hormone for skin coating treatment, but the long-term use of hormone therapy is greatly possible to trigger dyslipidemia, dysarteriotony and glucose abnormality, also lead to the excessive loss of calcium (5, 6). It is urgent to find a new and effective therapy for AD treatment with less even no side effects.

Cold atmospheric plasma (CAP) is a sort of ionized gas at the room temperature level that is composed of massive active particles like ions, electrons, free radicals, reactive oxygen species (ROS) and nitrogen species (7). CAP has been widely involved in medical applications. CAP is able to promote acute and chronic wound healing, improve oral cleaning and disinfection, facilitate cancer treatment (8–10). It has been demonstrated that CAP up-regulates the expression of hypoxia-inducible factor-1 (HIF-1) in human dermal fibroblasts (11, 12). HIF-1 is a heterodimer consisting of HIF-1α and HIF-1β monomers, which has been found to be highly expressed in skin injury, hypoxia and radiotherapy response (13, 14).

Mesencephalic astrocyte-derived neurotrophic factor (MANF) is a member of neurotrophic factor family to exert the protective effect on neurons and some non-neuronal cells (15–18). Also, in response to ER stress, MANF expression is up-regulated as one of ER stress-related proteins (19). Recently, more research evidences have demonstrated that MANF plays an anti-inflammatory role in some acute and chronic inflammatory diseases (17, 18, 20), which is mediated by binding to NF-κB p65 for impeding p65 nuclear translocation, further negatively affecting NF-κB signal activation (21). Although MANF’s inflammation inhibitory effect has been clearly verified, there is still no experimental finding to define the relationship between MANF and skin inflammation.

In this study, dinitrofluorobenzene (DNFB)-induced AD mice model was constructed to explore the effects of CAP and MANF on AD progress. Moreover, using human immortal keratinocyte line (HaCaT) *in vitro*, the transcriptional regulatory mechanism of HIF-1α induced by CAP on MANF expression was studied. These research results suggest the clinical application potential of CAP on AD treatment and prevention, also preliminarily reveal HIF-1α-mediated MANF transcriptional regulation.

## Method details

### DNFB-Induced AD Mice Model

6-8 weeks C57BL/6J mice were depilated on the skin of back (Area: 3 cm^2^). 0.5% DNFB in the mixed solution of acetone and olive oil (3:1) was used for coating mice’s depilated area every three days for four times. For CAP treatment, DNFB-induced AD mice were treated by CAP for 3 minutes. For hrMANF or MANF antibody treatment, DNFB-induced AD mice were injected subcutaneously by hrMANF protein (0.5 mg/kg) or MANF antibody (600μg/kg). Mice breeding was operated in SPF-class animal laboratory. All animal experiments were performed according to protocols approved by the Animal Ethics Committee of Anhui Medical University (Approval number: LLSC20210791).

### HaCaT Cell Culture and CAP Treatment

The human immortal keratinocyte HaCaT cell line was cultured in Dulbecco’s Modified Eagle’s Medium (DMEM) with 10% fetal bovine serum (FBS). The culture condition was 37°C and 5% CO_2_. CAP was produced by an atmospheric pressure dielectric barrier discharge jet plasma source mainly consisting of a quartz tube (inner diameter 4 mm) and a cooper ring. The cooper ring was powered by a high-voltage power supply generating a sinusoidal voltage waveform with 8.25 kV peak value at a frequency of 10 kHz. Helium (flow rate: 400 sccm) and oxygen (flow rate: 4 sccm) were mixed to introduce into the quartz tube. For mice skin treatment, the skin inflammation area was involved in CAP treatment for 3 minutes. For HaCaT cell treatment *in vitro*, HaCaT cell nutrient solution was firstly treated by CAP for 30 seconds, which was next used to cultivate HaCaT cells for overnight.

### Antibodies and Reagents

Antibodies involved in this study contain: anti-Cleaved caspase3 (Abcam, ab32042); anti-HMGB1 (Abcam, ab79823); anti-TNF-α (Abcam, ab183218); anti-IL-1β (Abcam, ab9722); anti-CCL2 (Abcam, ab25124); anti-Bip (Abcam, ab21685); anti-CHOP (Abcam, ab11419); anti-HO-1 (Abcam, ab52947); anti-MANF (Abcam, ab67271); anti-GAPDH (Abcam, ab3285); anti-HIF-1α (Abcam, ab243860); Anti-CD163 (Abcam, ab182422); Goat Anti-Rabbit IgG H&L (HRP) (Abcam, ab6721); PE anti-CD11b (Abcam, ab25533); APC anti-Ly6C (Abcam, ab93550); Alexa Flour 488 anti-Ly6G (Abcam, ab283276). The involved reagents contain: DNFB (Sigma, St Louis, MO, USA, 42085); hrMANF protein (Abcam, ab123227); Goat Anti-Mouse/Rabbit Polymer Immunohistochemistry Detection Kit (ZSGB-BIO, PV-6000); Lipofectamine™ 3000 (Thermo Fisher, L3000150).

### Immunohistochemistry

Mice’s skin tissues (n=5) were used for Immunohistochemistry according to the previous research (20). Skin tissues were fixed in 10% formaldehyde. Paraffin sections were produced after paraffin embedding, then deparaffinization in dimethylbenzene. Rehydration was performed in 100%, 90%, 80% and 70% ethanol for 5 minutes respectively. Hematoxylin and eosin were used for hematoxylin-eosin (HE) staining. After rinse, paraffin sections were performed by tissue antigen recovery, followed by heating and 1×PBS rinse. Peroxidase blocking agent was used for incubation at 37°C for 30 minutes. After 1×PBS rinse, paraffin sections were incubated with the goat serum at 37°C for 30 minutes. Then, the corresponding antibodies were used for incubation at 4°C overnight. After secondary antibody incubation at 37°C for 30 minutes and 1×PBS rinse, 3, 3′-diaminobenzidinetetrahydrochloride (DAB) and hematoxylin staining were performed. For immumohistochemical staining, paraffin sections were stained by antibodies of Cleaved caspase3 (1/300), HMGB1 (1/300), TNF-α (1/400), IL-1β (1/400), CCL2 (1/400), Bip (1/500), CHOP (1/300), HO-1 (1/1000), MANF (1/200), CD163 (1/400) and HIF-1α (1/400). Images were obtained by Olympus Microscope BX53.

### Western Blot

The reduced sodium dodecyl sulfate polyacrylamide gel electrophoresis (SDS-PAGE, 12%) was performed to separate protein samples extracted from mice’s skin tissues (n=5) and cells. Each protein sample was 10 μg. After SDS-PAGE, PVDF membrane (0.45μm, 26.5 cm x 3.75 m) was used for protein transfer, followed by 5% BSA, primary antibodies (anti-Cleaved caspase3, 1/500; anti-HMGB1, 1/20000; anti-TNF-α, 1/1000; anti-IL-1β, 1/1000; anti-CCL2, 1/1000; anti-Bip, 1/2000; anti-CHOP, 1/800; anti-HO-1, 1/2000; anti-MANF, 1/1000; anti-GAPDH, 1/5000; anti-HIF-1α, 1/1000; anti-CD163, 1/1000) and second antibodies (1/4000) incubation. Images were obtained by Chemiscope 6000 pro touch imaging system.

### Real Time-Quantitative Polymerase Chain Reaction

Total RNA extraction from mice’s skin tissues (n=5) was obtained by Trizol reagent, and reverse transcription was performed by PrimeScript RT reagent Kit (TaKaRa Bio, Dalian, China) according to manufacturer’s instruction. The involved primers contain: TNF-α, forward 5′-CAGGAGGGAGAACAGAAACTCCA-3′ and reverse 5′-CCTGGTTGGCTGCTTGCTT-3′; HMGB1, forward 5′-GCTGACAAGGCTCGTTATGAA-3′ and reverse 5′-CCTTTGATTTTGGGGCGG

TA-3′; IL-1β, forward 5′-GAAATGCCACCTTTTGACAGTG-3′ and reverse 5′-TGGATGCTCTCATCAGGACAG-3′; CCL2, forward 5′-TAAAAACCTGGATCG

GAACCAAA-3′ and reverse 5′- GCATTAGCTTCAGATTTACGGGT-3′; Bip, forward 5′-ACTTGGGGACCACCTATTCCT-3′ and reverse 5′-GTTGCCCTGATCG

TTGGCTA-3′; CHOP, forward 5′- AAGCCTGGTATGAGGATCTGC-3′ and reverse 5′-TTCCTGGGGATGAGATATAGGTG-3′; HO-1, forward 5′-AGGTACACATCCAA

GCCGAGA-3′ and reverse 5′-CATCACCAGCTTAAAGCCTTCT-3′; MANF, forward 5′-TCTGGGACGATTTTACCAGGA-3′ and reverse 5′-CTTGCTTCACGGC

AAAACTTT-3′; CD163, forward 5′-GGTGGACACAGAATGGTTCTTC-3′ and reverse 5′-CCAGGAGCGTTAGTGACAGC-3′; GAPDH, forward 5′-AGGTCGGTG

TGAACGGATTTG-3′ and reverse 5′-GGGGTCGTTGATGGCAACA-3′. The 2^-ddCT calculation was used.

### Enzyme-Linked Immunosorbent Assay

The serum from mice (n=8) was collected to examine the serum levels of TNF-α, IL-1β and IL-10. The involved ELISA kits contain: Mouse TNF-α *in vitro* SimpleStep ELISA Kit (Abcam, ab208348); Mouse IL-1 beta *in vitro* SimpleStep ELISA Kit (Abcam, ab100704); Mouse IL-10 *in vitro* SimpleStep ELISA Kit (Abcam, ab255729). ELISA was performed according to manufacturer’s instructions.

### Terminal Deoxynucleotidyl Transferase dUTP Nick end Labeling Assay

Paraffin-embedded mice skin tissues (n=5) were prepared for TUNEL assay. *In Situ* Cell Death Detection Kit, Fluorescein (Roche, Basel, Switzerland, 11684795910) was used according to manufacturer’s instruction. The final results were acquired by Olympus Microscope BX53/IX71.

### ROS and NO Examination

The serum from mice (n=8) was collected to examine the serum ROS and NO levels. Total Reactive Oxygen Species and Nitric Oxide Assay Kit (Nanjing Jiancheng Bioengineering Institute, Nanjing, Jiangsu, China) was used for ROS and NO examination according to manufacturer’s instruction.

### Flow Cytometry

Peripheral blood samples from mice were used for flow cytometry assay. Cells in peripheral blood were blocked by 1% mouse serum, then antibody incubation was performed for 30 minutes. After PBS washing, CD11b^+^Ly6C^hi^ and CD11b^+^Ly6G^+^ immune cells in peripheral blood were analyzed by BD FACS Verse.

### Chromatin Immunoprecipitation

HaCaT cells were processed according to previous protocol (22). Anti-HIF-1α and normal IgG antibodies were separately added into lysates. After CHIP assay, PCR was performed for HIF-1α-MANF promoter binding analysis. Human MANF-pro-HIF-1α primers: forward 5’-CAACGGTTCCCGCATCCTG-3’ and reverse 5’-CTGAATCGTGGCTTGGTGG-3’.

### Dual-Luciferase Reporter Assey

HaCaT cells were co-transfected with luciferase reporters of pGL3-MANF promoter control or pGL3-MANF promoter HIF-1α binding site mutation plasmid together with pcDNA-control or pcDNA-HIF-1α mutation plasmid, followed by cell culture for 24 hours. Cell lysate was extracted to examine the luciferase activity by Dual-Luciferase Reporter Assay System (Promega, USA). Renilla luciferase activity was used for normalization.

### Statistical Analysis

Data are presented as means ± SD. Two-way ANOVA was used for statistical comparison. *p value*<0.05 indicates significant difference. An asterisk (*), two asterisks (**) and three asterisks (***) stand for *p*<0.05, *p*<0.01 and *p*<0.001 respectively. For mice experiments, 8 mice per group (n=8) were used. All experiments were performed independently at least three times.

## Results

### CAP Treatment Weakened DNFB-Induced Apoptosis to Relieve Skin Injury in Mice

**Figure 1:**
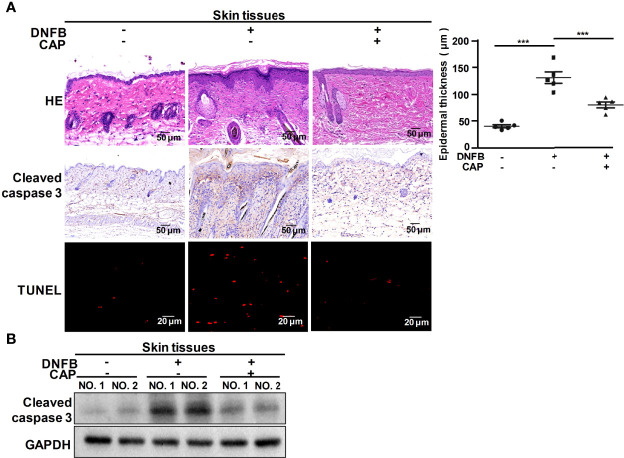
CAP alleviated DNFB-induced skin injury *via* attenuating apoptosis. DNFB-induced AD mice model was constructed, followed by CAP treatment, n=8. Skin tissues (n=5) were used for HE, immunohistochemical staining of cleaved caspase 3 and TUNEL assay **(A)**, as well as western blot of cleaved caspase 3 **(B)**. Epidermal thickness was evaluated. GAPDH serves as control for normalization. Data are expressed as mean ± SEM. ***p < 0.001.

To clarify the effect of CAP on AD, we constructed DNFB-induced AD mice model according to the previous reports (23, 24). **Figure 1A** showed that DNFB was able to promote skin thickening, induce hyperkeratosis and parakeratosis, increase the skin tissue infiltration of inflammatory cells. Comparatively, although CAP treatment alone could not induce skin injury and cell apoptosis in mice (**Figures S1A, B**), CAP treatment greatly weakened DNFB-induced skin injury in mice (**Figure 1A**). Caspase 3 is one of the classic apoptosis-associated proteins (25). IHC and WB results showed that cleaved caspase 3 was remarkably up-regulated in DNFB-induced mice skin tissues. After CAP treatment, the increase of cleaved caspase 3 induced by DNFB was restrained (**Figures 1A, B** and **S1C, D**). Moreover, TUNEL assay results in **Figures 1A** and **S1C** showed that CAP treatment was able to decrease DNFB-induced skin cell apoptosis. These evidences suggest CAP treatment relieves DNFB-induced apoptosis in skin tissues of mice, finally decreasing DNFB-induced skin injury.

### CAP Treatment Attenuated DNFB-Induced Skin Inflammation, ER Stress and Oxidative Stress in Mice

**Figure 2:**
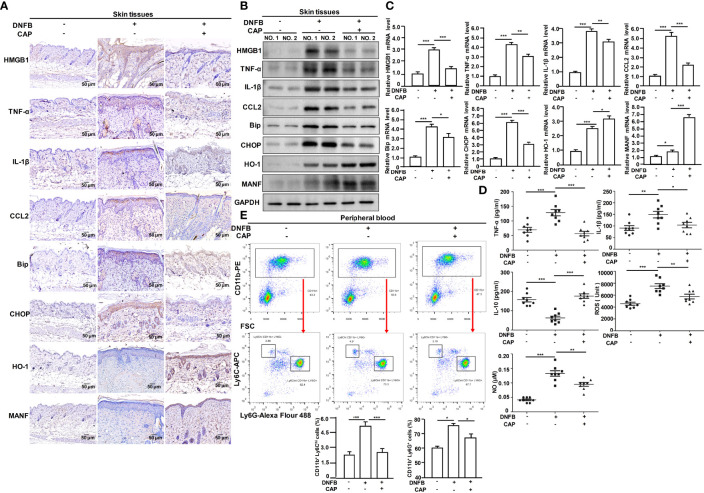
CAP reduced DNFB-induced skin inflammation, ER stress and oxidative stress in mice. DNFB-induced AD mice model was constructed, followed by CAP treatment, n=8. Skin tissues (n=5) were used for immunohistochemical staining of HMGB1, TNF-α, IL-1β, CCL2, Bip, CHOP, HO-1 and MANF **(A)**, as well as western blot **(B)** and RT-qPCR **(C)** of the indicated proteins. GAPDH serves as control for normalization. **(D)** Serum samples (n=8) were used for ELISA of TNF-α, IL-1β, IL-10, NO and ROS. **(E)** CD11b^+^Ly6C^hi^ and CD11b^+^Ly6G^+^ cells were examined by flow cytometry. Data are expressed as mean ± SEM. *p < 0.05, **p < 0.01, *** p < 0.001.

The inflammatory response is often accompanied by ER stress and oxidative stress responses (26). Accordingly, we examined the commonly-used indicators of inflammation, ER stress and oxidative stress, including Tumor Necrosis Factor-α (TNF-α), Interleukin-1β (IL-1β) (27), High Mobility Group Box 1 (HMGB1) (28), Chemokine CCL2 (29), Glucose Regulated Protein 78 (GRP78, also known as Bip), CCAAT/enhancer binding protein homologous protein (CHOP) (30), Heme Oxygenase-1 (HO-1) (31) and MANF. IHC, WB and RT-qPCR results showed that DNFB stimulation could promote expressions of pro-inflammatory cytokines TNF-α and IL-1β, Chemokine CCL2, pro-inflammatory HMGB1, ER stress-related proteins Bip and CHOP, oxidative stress-related protein HO-1 in skin tissues of mice, indicating DNFB-induced AD mice had the greater inflammation, ER stress and oxidative stress responses compared with untreated mice; also, CAP treatment partly alleviated DNFB-induced skin inflammation, ER stress and oxidative stress (**Figures 2A–C** and **S2A, B**). Consistently, DNFB-induced AD mice had the higher serum levels of TNF-α, IL-1β, ROS and NO, but the lower serum anti-inflammatory IL-10. CAP treatment significantly inhibited the serum TNF-α, IL-1β, ROS and NO, also promoted the serum IL-10 (**Figure 2D**). In the AD process, it has been found that pro-inflammatory immune cells were increased in peripheral blood (32, 33). As shown in **Figure 2E**, after DNFB stimulation, the proportions of CD11b^+^Ly6C^hi^ monocytes and CD11b^+^Ly6G^+^ neutrophils in peripheral blood of mice were greatly increased, which were inversely lowered by CAP treatment. By M2-type macrophage marker CD163 detection in **Figure S2C and D**, the skin M2 macrophage differentiation was greatly promoted by CAP treatment in DNFB-induced AD mice. Moreover, we found that DNFB slightly stimulated MANF transcription and expression in skin tissues of mice, which was further greatly promoted by CAP treatment (**Figures 2A–C** and **S2A, B**). The above data suggest that DNFB-induced skin inflammation, ER stress and oxidative stress are weakened by CAP treatment that largely up-regulates MANF expression.

### CAP Treatment Enhanced MANF Expression to Reduce DNFB-Induced Skin Inflammatory Injury, ER Stress and Oxidative Stress in Mice

**Figure 3:**
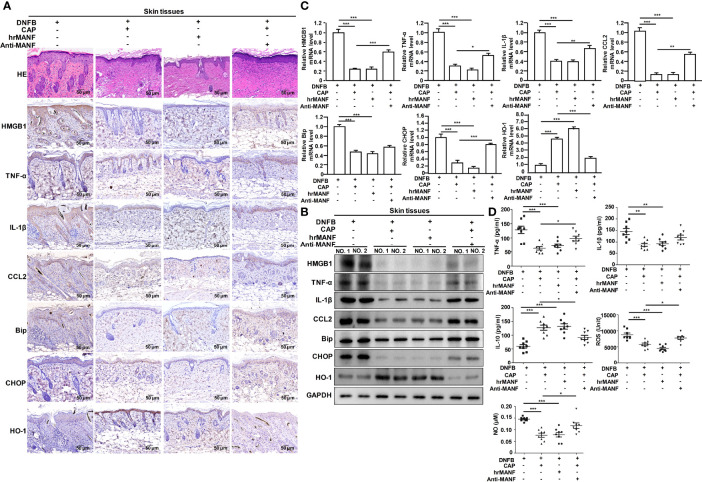
CAP relieved DNFB-induced skin inflammatory injury, ER stress and oxidative stress *via* promoting MANF expression. DNFB-induced AD mice model was constructed, followed by CAP treatment, hrMANF treatment and MANF antibody treatment, n=8. Skin tissues (n=5) were used for HE and immunohistochemical staining of HMGB1, TNF-α, IL-1β, CCL2, Bip, CHOP and HO-1 **(A)**, as well as western blot **(B)** and RT-qPCR **(C)** of the indicated proteins. GAPDH serves as control for normalization. **(D)** Serum samples (n=8) were used for ELISA of TNF-α, IL-1β, IL-10, NO and ROS. Data are expressed as mean ± SEM. *p < 0.05, **p < 0.01, *** p < 0.001.

Next, we studied whether CAP treatment suppressed AD occurrence and development *via* MANF up-regulation. The exogenous human recombinant MANF (hrMANF) protein and MANF antibody were used to treat DNFB-induced AD mice. The treatment of CAP, hrMANF and MANF antibody alone did not significantly affect skin integrity, inflammation, ER stress and oxidative stress of mice (**Figures S3A–C**). As shown in **Figures 3A–D** and **S4A, B**, hrMANF treatment significantly relieved DNFB-induced skin inflammation, ER stress and oxidative stress, which was consistent with the effect of CAP treatment. However, inhibiting MANF protein *via* MANF antibody could partly resist the protective effect of MANF against DNFB-induced skin inflammation injury, ER stress and oxidative stress in mice, indicating the protective effect of CAP against DNFB-induced skin inflammation injury was mediated by CAP-induced MANF up-regulation. Therefore, MANF expression induced by CAP treatment plays an important role in CAP-mediated inhibitory effect on DNFB-induced skin inflammation, ER stress and oxidative stress responses.

### CAP treatment Induced MANF Transcriptional Expression *via* Increasing HIF-1α Level

**Figure 4:**
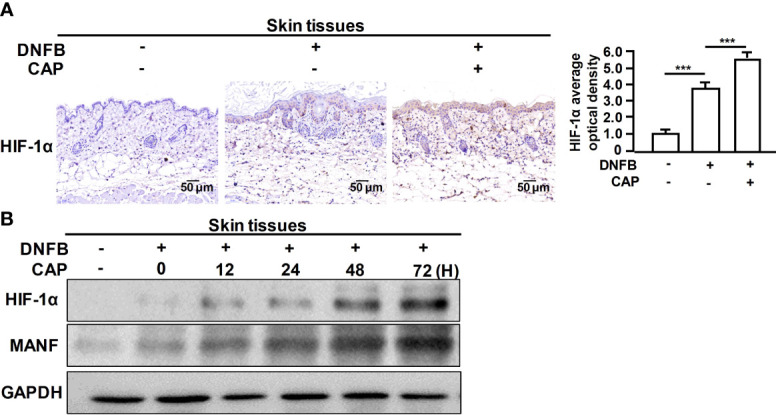
CAP treatment significantly enhanced DNFB-induced HIF-1α expression in skin tissues. DNFB-induced AD mice model was constructed, followed by CAP treatment, n=8. **(A)** Skin tissues (n=5) were used for immunohistochemical staining of HIF-1α. The average optical density was analyzed. **(B)** At 0, 12, 24, 48 and 72 hours after CAP treatment, skin tissues (n=5) were used for western blot of HIF-1α and MANF. GAPDH serves as control for normalization. Data are expressed as mean ± SEM. ***p < 0.001.

**Figure 5:**
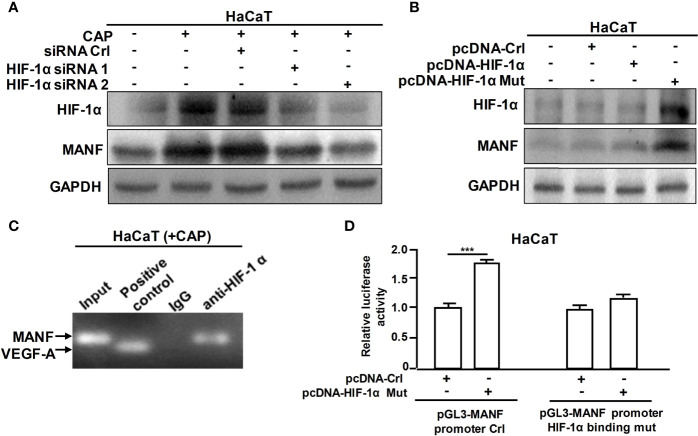
CAP induced MANF transcription and expression by HIF-1α-mediated transcriptional regulation. **(A)** HaCaT cells transfected by siRNA control, HIF-1α siRNA 1 and HIF-1α siRNA 2 respectively were treated by CAP, followed by western blot of HIF-1α and MANF. **(B)** HaCaT cells transfected by pcDNA-control, pcDNA-HIF-1α and pcDNA-HIF-1α mutation plasmid respectively were treated by CAP, followed by western blot of HIF-1α and MANF. GAPDH serves as control for normalization. **(C)** HaCaT cells treated by CAP were used for Chromatin Immunoprecipitation assay. HIF-1α antibody was used for HIF-1α protein immunoprecipitation. IgG antibody serves as negative control. VEGF-A was involved as a positive control for HIF-1α binding. **(D)** HaCaT cells transfected by pcDNA-control and pcDNA-HIF-1α mutation plasmid respectively were used for dual-luciferase reporter assay of pGL3-MANF promoter control or pGL3-MANF promoter HIF-1α binding site mutation plasmid. The relative luciferase activity was analyzed. All experiments were performed independently at least three times. Data are expressed as mean ± SEM. ***p < 0.001.

Furthermore, we explored the specific mechanism on CAP-mediated MANF transcriptional regulation. It has been reported that CAP is able to increase HIF-1α expression (11). We also found that CAP treatment alone could slightly increase HIF-1α and MANF levels in skin tissues of mice (**Figures S5A, B**). We have previously found that there is a potential HIF-1α binding site in the promoter region of human *manf* gene (From +357 to +365), suggesting the possibility of HIF-1α-mediated direct transcriptional regulation for MANF expression. IHC results in **Figure 4A** showed that DNFB stimulated HIF-1α expression, and CAP treatment further increased HIF-1α level in skin tissues of mice. In **Figure 4B** and **Figure S5C**, CAP treatment could significantly promote HIF-1α and MANF expressions in skin tissues of mice in a time-dependent way. To clarify the interplay among CAP, HIF-1α and MANF expression, we performed a series of experiments *in vitro* by using HaCaT cells. We conducted HIF-1α gene silencing by two different HIF-1α siRNA sequences in HaCaT cells. After HIF-1α expression was down-regulated, the intracellular MANF level was decreased consequently (**Figure 5A** and **Figure S5D**). Also, HIF-1α mutant protein expression plasmid (pcDNA-HIF-1α Mut) was constructed to restrain HIF-1α degradation. As shown in **Figure 5B** and **Figure S5E**, after HIF-1α mutant protein was over-expressed in HaCaT cells, MANF expression was greatly promoted. These data indicate there is a positive correlation between HIF-1α and MANF expression in both mice skin tissues and HaCaT cells. CHIP result in **Figure 5C** showed that HIF-1α could bind to *manf* gene promoter region after CAP treatment. Over-expression of HIF-1α mutant protein was able to markedly enhance *manf* promoter’s activity, but HIF-1α binding site mutation in *manf* promoter eliminated HIF-1α-mediated MANF transcriptional activation (**Figure 5D**). Altogether, CAP treatment induces the expression of HIF-1α that directly binds to *manf* promoter region for MANF transcriptional activation.

## Discussion

Atopic dermatitis is a sort of common skin disease with the characteristic of chronic inflammation to show the systemic disorder, further progressing to asthma, allergic rhinitis and other diseases (34). DNFB is a commonly-used chemical reagent to induce AD-like skin inflammation and injury (35). There are some previous reports and researches that use DNFB-induced mouse skin inflammation as allergic contact dermatitis and atopic dermatitis models (36–39). In this study, we used DNFB-induced AD mice model to reveal the effect of CAP on the pathological process of AD. Our findings indicate that CAP exerts an inhibitory effect on DNFB-induced AD-like skin inflammatory injury, ER stress and oxidative stress responses in mice. Currently, the clinical therapy for AD is mainly based on corticosteroid hormone, which gives rise to some side effects (6). In light of CAP’s negative effect on AD, it is potential to involve CAP in the clinical treatment of AD without significant side effects. In our previous research (40), we have found that CAP is able to decrease the human non-small cell lung carcinoma A549 cell inflammation and oxidant stress induced by Tunicamycin. The transitory and low-intensity CAP treatment only induces a degree of ROS increase, not the overwhelming ROS production. Consistently, we speculate that the slight ROS production induced by CAP in our study may exert the anti-inflammatory and antioxidative effect *via* amplifying the correlated signaling pathways.

For the mechanism on how CAP restrains DNFB-induced skin inflammation and injury, our study suggests CAP-induced MANF expression in skin tissues plays a key role to mediate CAP’s protective effect against DNFB-induced AD in mice. Without CAP treatment, DNFB stimulation purely is able to slightly increase MANF level in skin tissues. Interestingly, MANF transcriptional expression is significantly promoted by CAP treatment. MANF has been proven to exert the anti-inflammatory effect in multiple inflammation-linked diseases, like acute kidney injury (17), bacterial myocarditis (18) and antigen-induced arthritis (21). This study further expands MANF’s anti-inflammatory role in atopic dermatitis, possibly other skin inflammatory diseases. In addition, there are some researches demonstrating MANF’s moderating effects on functional differentiation of macrophages (41). Overall, macrophages are mainly divided into two different functional subtypes: pro-inflammatory M1 macrophages and anti-inflammatory M2 macrophages (42). Joana Neves et al. have found that MANF is able to induce YM^+^Arg^+^ M2 anti-inflammatory macrophage polarization in an autocrine way for retinal damage repair (41). Also, mono-macrophage-specific MANF deficiency significantly affects M1/M2 differentiation of splenic macrophages in the hepatic fibrosis process (20). We examined pro-inflammatory immune cells in peripheral blood of mice, then found that CAP treatment could partly suppress the increased proportion of pro-inflammatory CD11b^+^Ly6C^hi^ monocytes and CD11b^+^Ly6G^+^ neutrophils in peripheral blood induced by DNFB stimulation, as well as promote M2 anti-inflammatory macrophage differentiation *via* CD163 detection. In the future, we plan to analyze the change of macrophages’ M1/M2 differentiation in skin tissues and peripheral blood of mice after CAP treatment. Besides MANF, there are many other target genes transcriptionally regulated by HIF-1α, including some anti-apoptosis genes (43), which may mediate CAP’s protective effect on DNFB-induced AD.

As an ER stress-related protein, MANF has been demonstrated to be up-regulated *via* the direct binding of XBP1s to ER stress response elements in MANF promoter region (44). In this study, we found that HIF-1α had a direct transcriptional regulation on MANF expression, and there was a verified HIF-1α binding site in MANF promoter. The previous researches have reported that MANF expression in glial cells is enhanced under the condition of focal cerebral ischemia (45); also, ischemia in heart is able to induce MANF expression as well (46). These evidences indicate that the ischemic and hypoxic environment contributes to MANF up-regulation, which may be attributed to ER stress response induced by ischemia-hypoxia (45–47). Therefore, two different pathways are involved in hypoxia-caused MANF up-regulation. Unfolded protein response (UPR) is often intensified by hypoxia to indirectly enhance MANF expression. Moreover, hypoxia improves HIF-1α protein stability *via* inhibition of HIF-1α degradation to increase the intracellular HIF-1α level (48), further promote HIF-1α-mediated MANF transcriptional expression. Besides hypoxia, our data showed that CAP treatment could raise HIF-1α level in the non-hypoxic condition. It has been found that mitochondrial-derived ROS is a non-hypoxic factor for HIF-1α stabilization and HIF-1 activation (49, 50). It is possible that ROS generated by CAP irradiation leads to the increase of HIF-1α in the non-hypoxic environment.

## Conclusion

In this study, CAP treatment is able to protect against DNFB-induced skin inflammation, ER stress and oxidative stress of mice, further alleviate DNFB-induced mice skin injury. The protective effect of CAP on DNFB-induced AD mice model is mediated by CAP-induced MANF up-regulation. CAP promotes the increase of HIF-1α that binds to MANF promoter region for MANF transcriptional activation and expression.

## Data Availability Statement

The original contributions presented in the study are included in the article/supplementary material. Further inquiries can be directed to the corresponding authors.

## Ethics Statement

The animal study was reviewed and approved by School of Basic Medical Sciences, Anhui Medical University, Hefei, China. Written informed consent was obtained from the owners for the participation of their animals in this study.

## Author Contributions

GN, DW and LF designed the research and wrote the manuscript. TS, XZ, CH, SY, LK, CL, YZ, and ZY performed the experiments, collected and analyzed the data. All authors contributed to the article and approved the submitted version.

## Funding

The study was supported by the fund from National Key R&D Program of China to Guohua Ni (Grant number: 2019YFC0119000), National Natural Science Foundation of China to DW and GN (Grant number: 31800702, 11875295 and 11535003), Funds from Anhui Medical University to DW (Grant number: XJ201603 and 2017xkj003).

## Conflict of Interest

The authors declare that the research was conducted in the absence of any commercial or financial relationships that could be construed as a potential conflict of interest.

## Publisher’s Note

All claims expressed in this article are solely those of the authors and do not necessarily represent those of their affiliated organizations, or those of the publisher, the editors and the reviewers. Any product that may be evaluated in this article, or claim that may be made by its manufacturer, is not guaranteed or endorsed by the publisher.

## Abbreviations

AD, Atopic dermatitis; CAP, Cold atmospheric plasma; MANF, Mesencephalic astrocyte-derived neurotrophic factor; HIF-1, Hypoxia-inducible factor-1; DNFB, dinitrofluorobenzene; TM, Tunicamycin; ROS, Reactive oxygen species; NF-κB, Nuclear factor kappa-B.

## References

[1] Leung DYM Boguniewicz M Howell MD Nomura I Hamid OA. New Insights Into Atopic Dermatitis. J Clin Invest (2004) 113(5):651–7. doi: 10.1172/jci200421060 PMC351324 14991059

[2] Toncic RJ Marinovic B. The Role of Impaired Epidermal Barrier Function in Atopic Dermatitis. Acta Dermatovenerologica Croatica (2016) 24(2):95–109. 27477169

[3] Robida PA Chumanevich AP Gandy AO Fuseler JW Nagarkatti P Nagarkatti M. Skin Mast Cell-Driven Ceramides Drive Early Apoptosis in Pre-Symptomatic Eczema in Mice. Int J Mol Sci (2021) 22(15):7851. doi: 10.3390/ijms22157851 34360617 PMC8346072

[4] Ji HX Li XK. Oxidative Stress in Atopic Dermatitis. Oxid Med Cell Longev (2016) 2016:2721469. doi: 10.1155/2016/2721469 27006746 PMC4781995

[5] Clore JN Thurby-Hay L. Glucocorticoid-Induced Hyperglycemia. Endocrine Pract (2009) 15(5):469–74. doi: 10.4158/ep08331.rar 19454391

[6] Oray M Abu Samra K Ebrahimiadib N Meese H Foster CS. Long-Term Side Effects of Glucocorticoids. Expert Opin Drug Saf (2016) 15(4):457–65. doi: 10.1517/14740338.2016.1140743 26789102

[7] Brany D Dvorska D Halasova E Skovierova H. Cold Atmospheric Plasma: A Powerful Tool for Modern Medicine. Int J Mol Sci (2020) 21(8):2932. doi: 10.3390/ijms21082932 PMC7215620 32331263

[8] Duarte S Panariello BHD. Comprehensive Biomedical Applications of Low Temperature Plasmas. Arch Biochem Biophys (2020) 693:108560. doi: 10.1016/j.abb.2020.108560 32857998 PMC7448743

[9] Heinlin J Morfill G Landthaler M Stolz W Isbary G Zimmermann JL. Plasma Medicine: Possible Applications in Dermatology. J Der Deutschen Dermatologischen Gesellschaft (2010) 8(12):968–76. doi: 10.1111/j.1610-0387.2010.07495.x 20718902

[10] Setsuhara Y. Low-Temperature Atmospheric-Pressure Plasma Sources for Plasma Medicine. Arch Biochem Biophys (2016) 605:3–10. doi: 10.1016/j.abb.2016.04.009 27109191

[11] Cui HS Joo SY Lee DH Yu JH Jeong JH Kim JB. Low Temperature Plasma Induces Angiogenic Growth Factor *Via* Up-Regulating Hypoxia-Inducible Factor 1 Alpha in Human Dermal Fibroblasts. Arch Biochem Biophys (2017) 630:9–17. doi: 10.1016/j.abb.2017.07.012 28750820

[12] Lee HY Lee HJ Kim GC Choi JH Hong JW. Plasma Cupping Induces Vegf Expression in Skin Cells Through Nitric Oxide-Mediated Activation of Hypoxia Inducible Factor 1. Sci Rep (2019) 9:3821. doi: 10.1038/s41598-019-40086-8 30846730 PMC6405951

[13] Chen L Gajendrareddy PK DiPietro LA. Differential Expression of Hif-1 Alpha in Skin and Mucosal Wounds. J Dent Res (2012) 91(9):871–6. doi: 10.1177/0022034512454435 PMC3420394 22821237

[14] Dewhirst MW Cao Y Moeller B. Cycling Hypoxia and Free Radicals Regulate Angiogenesis and Radiotherapy Response. Nat Rev Cancer (2008) 8(6):425–37. doi: 10.1038/nrc2397 PMC3943205 18500244

[15] Apostolou A Shen Y Liang Y Luo J Fang S. Armet, a Upr-Upregulated Protelin, Inhibits Cell Proliferation and Er Stress-Induced Cell Death. Exp Cell Res (2008) 314(13):2454–67. doi: 10.1016/j.yexcr.2008.05.001 PMC6719340 18561914

[16] Yang W Shen Y Chen Y Chen L Wang L Wang H. Mesencephalic Astrocyte-Derived Neurotrophic Factor Prevents Neuron Loss Via Inhibiting Ischemia-Induced Apoptosis. J Neurol Sci (2014) 344(1-2):129–38. doi: 10.1016/j.jns.2014.06.042 25001514

[17] Hou C Mei Q Song X Bao Q Li X Wang D. Mono-Macrophage-Derived Manf Protects Against Lipopolysaccharide-Induced Acute Kidney Injury *Via* Inhibiting Inflammation and Renal M1 Macrophages. Inflammation (2020) 44(2):693–703. doi: 10.1007/s10753-020-01368-w 33145627

[18] Wang C Bao Q Hou C Sun M Song X Cao S. Mono-Macrophage-Derived Manf Alleviates Bacterial Myocarditis by Inhibiting Nf-Kappab Activation and Myocardial Inflammation. Inflammation (2021) 44(5):1916–26. doi: 10.1007/s10753-021-01469-0 33939070

[19] Kim Y Park SJ Chen YM. Mesencephalic Astrocyte-Derived Neurotrophic Factor (Manf), a New Player in Endoplasmic Reticulum Diseases: Structure, Biology, and Therapeutic Roles. Transl Res (2017) 188:1–9. doi: 10.1016/j.trsl.2017.06.010 28719799 PMC5601018

[20] Hou C Wang D Li X He Y Wei C Jiang R. Manf Regulates Splenic Macrophage Differentiation in Mice. Immunol Lett (2019) 212:37–45. doi: 10.1016/j.imlet.2019.06.007 31226359

[21] Chen L Feng L Wang X Du J Chen Y Yang W. Mesencephalic Astrocyte-Derived Neurotrophic Factor Is Involved in Inflammation by Negatively Regulating the Nf-Kappa B Pathway. Sci Rep (2015) 5:8133. doi: 10.1038/srep08133 25640174 PMC4313098

[22] Ren ZJ Kang WY Wang LH Sun BL Ma JJ Zheng CG. E2f1 Renders Prostate Cancer Cell Resistant to Icam-1 Mediated Antitumor Immunity by Nf-Kb Modulation. Mol Cancer (2014) 13:84. doi: 10.1186/1476-4598-13-84 24742333 PMC4004456

[23] Kim TH Jung JA Kim GD Jang AH Ahn HJ Park YS. Melatonin Inhibits the Development of 2,4-Dinitrofluorobenzene-Induced Atopic Dermatitis-Like Skin Lesions in Nc/Nga Mice. J Pineal Res (2009) 47(4):324–9. doi: 10.1111/j.1600-079X.2009.00718.x 19817972

[24] Shi YL Gu J Park JY Xu YP Yu FS Zhou L. Histone Deacetylases Inhibitor Trichostatin a Ameliorates Dnfb-Induced Allergic Contact Dermatitis and Reduces Epidermal Langerhans Cells in Mice. J Dermatol Sci (2012) 68(2):99–107. doi: 10.1016/j.jdermsci.2012.09.001 22999682 PMC3482471

[25] Bernard A Chevrier S Beltjens FCC Dosset M Viltard E Lagrange A. Cleaved Caspase-3 Transcriptionally Regulates Angiogenesis-Promoting Chemotherapy Resistance. Cancer Res (2019) 79(23):5958–70. doi: 10.1158/0008-5472.can-19-0840 31611309

[26] Dandekar A Mendez R Zhang K. Cross Talk Between Er Stress, Oxidative Stress, and Inflammation in Health and Disease. Methods Mol Biol (Clifton NJ) (2015) 1292:205–14. doi: 10.1007/978-1-4939-2522-3_15 25804758

[27] Keum H Kim TW Kim Y Seo C Son Y Kim J. Bilirubin Nanomedicine Alleviates Psoriatic Skin Inflammation by Reducing Oxidative Stress and Suppressing Pathogenic Signaling. J Controlled Release (2020) 325:359–69. doi: 10.1016/j.jconrel.2020.07.015 32681946

[28] Yang H Wang HC Andersson U. Targeting Inflammation Driven by Hmgb1. Front Immunol (2020) 11:484. doi: 10.3389/fimmu.2020.00484 32265930 PMC7099994

[29] Qian BZ Li JF Zhang H Kitamura T Zhang JH Campion LR. Ccl2 Recruits Inflammatory Monocytes to Facilitate Breast-Tumour Metastasis. Nature (2011) 475(7355):222–U129. doi: 10.1038/nature10138 21654748 PMC3208506

[30] Sato N Urano F Yoon Leem J Kim SH Li M Donoviel D. Upregulation of Bip and Chop by the Unfolded-Protein Response Is Independent of Presenilin Expression. Nat Cell Biol (2000) 2(12):863–70. doi: 10.1038/35046500 11146649

[31] Chiang SK Chen SE Chang LC. The Role of Ho-1 and Its Crosstalk With Oxidative Stress in Cancer Cell Survival. Cells (2021) 10(9):2401. doi: 10.3390/cells10092401 34572050 PMC8471703

[32] Chan SC Shen K Gebhardt M Hanifin JM. The Role of Monocytes in Atopic Dermatitis Immunopathology. J Dermatol (2000) 27(11):696–7. doi: 10.1111/j.1346-8138.2000.tb02260.x 11138533

[33] Walsh CM Hill RZ Schwendinger-Schreck J Deguine J Brock EC Kucirek N. Neutrophils Promote Cxcr3-Dependent Itch in the Development of Atopic Dermatitis. eLife (2019) 8:33. doi: 10.7554/elife.48448 PMC6884397 31631836

[34] Boguniewicz M Leung DYM. Atopic Dermatitis. J Allergy Clin Immunol (2006) 117(2):S475–S80. doi: 10.1016/j.jaci.2005.10.018 16455350

[35] Lee JH Lee YS Lee EJ Lee JH Kim TY. Capsiate Inhibits Dnfb-Induced Atopic Dermatitis in Nc/Nga Mice Through Mast Cell and Cd4+T-Cell Inactivation. J Invest Dermatol (2015) 135(8):1977–85. doi: 10.1038/jid.2015.117 25806854

[36] Yuan XY Ma HM Li RZ Wang RY Liu W Guo JY. Topical Application of Aloperine Improves 2,4-Dinitrofluorobenzene-Induced Atopic Dermatitis-Like Skin Lesions in Nc/Nga Mice. Eur J Pharmacol (2011) 658(2-3):263–9. doi: 10.1016/j.ejphar.2011.02.013 21371468

[37] Heo JC Nam DY Seo MS Lee SH. Alleviation of Atopic Dermatitis-Related Symptoms by Perilla Frutescens Britton. Int J Mol Med (2011) 28(5):733–7. doi: 10.3892/ijmm.2011.763 21811759

[38] Han NR Moon PD Kim HM Jeong HJ. Effect of Pyeongwee-San (Kmp6) on 2,4-Dinitrofluorobenzene-Induced Atopic Dermatitis-Like Skin Lesions in Nc/Nga Mice. Life Sci (2012) 90(3-4):147–53. doi: 10.1016/j.lfs.2011.10.015 22075493

[39] Li WH Ding FM Zhai Y Tao WT Bi J Fan H. Il-37 Is Protective in Allergic Contact Dermatitis Through Mast Cell Inhibition. Int Immunopharmacol (2020) 83:106476. doi: 10.1016/j.intimp.2020.106476 32278131

[40] Sun T Yu SJ Song XG Zhang J Bao Q Mei Q. Cold Plasma Irradiation Regulates Inflammation and Oxidative Stress in Human Bronchial Epithelial Cells and Human Non-Small Cell Lung Carcinoma. Radiat Res (2022) 197(2):166–74. doi: 10.1667/rade-20-00178.1 34700340

[41] Neves J Zhu J Sousa-Victor P Konjikusic M Riley R Chew S. Immune Modulation by Manf Promotes Tissue Repair and Regenerative Success in the Retina. Science (2016) 353(6294):43. doi: 10.1126/science.aaf3646 PMC5270511 27365452

[42] Italiani P Boraschi D. From Monocytes to M1/M2 Macrophages: Phenotypical Vs. Functional Differentiation. Front Immunol (2014) 5:514. doi: 10.3389/fimmu.2014.00514 25368618 PMC4201108

[43] Wang XH Wei LL Li QC Lai YR. Hif-1 Alpha Protects Osteoblasts From Ros-Induced Apoptosis. Free Radical Res (2022) 56(2):143–53. doi: 10.1080/10715762.2022.2037581 35380485

[44] Wang D Hou C Cao Y Cheng Q Zhang L Li H. Xbp1 Activation Enhances Manf Expression *Via* Binding to Endoplasmic Reticulum Stress Response Elements Within Manf Promoter Region in Hepatitis B. Int J Biochem Cell Biol (2018) 99:140–6. doi: 10.1016/j.biocel.2018.04.007 29649564

[45] Shen Y Sun A Wang Y Cha D Wang H Wang F. Upregulation of Mesencephalic Astrocyte-Derived Neurotrophic Factor in Glial Cells Is Associated With Ischemia-Induced Glial Activation. J Neuroinflamm (2012) 9:254. doi: 10.1186/1742-2094-9-254 PMC3576245 23173607

[46] Tadimalla A Belmont PJ Thuerauf DJ Glassy MS Martindale JJ Gude N. Mesencephalic Astrocyte-Derived Neurotrophic Factor Is an Ischemia-Inducible Secreted Endoplasmic Reticulum Stress Response Protein in the Heart. CircRes (2008) 103(11):1249–58. doi: 10.1161/circresaha.108.180679 PMC2746824 18927462

[47] Diaz-Bulnes P Saiz ML Lopez-Larrea C Rodriguez RM. Crosstalk Between Hypoxia and Er Stress Response: A Key Regulator of Macrophage Polarization. Front Immunol (2020) 10:2951. doi: 10.3389/fimmu.2019.02951 31998288 PMC6961549

[48] Balamurugan K. Hif-1 at the Crossroads of Hypoxia, Inflammation, and Cancer. Int J Cancer (2016) 138(5):1058–66. doi: 10.1002/ijc.29519 PMC4573780 25784597

[49] Patten DA Lafleur VN Robitaille GA Chan DA Giaccia AJ Richard DE. Hypoxia-Inducible Factor-1 Activation in Nonhypoxic Conditions: The Essential Role of Mitochondrial-Derived Reactive Oxygen Species. Mol Biol Cell (2010) 21(18):3247–57. doi: 10.1091/mbc.e10-01-0025 PMC2938389 20660157

[50] Yoshida K Kirito K Hu Y Ozawa K Kaushansky K Komatsu N. Thrombopoietin (Tpo) Regulates Hif-1 Alpha Levels Through Generation of Mitochondrial Reactive Oxygen Species. Int J Hematol (2008) 88(1):43–51. doi: 10.1007/s12185-008-0091-6 18473128

