# Supplementary material for: Cold Plasma Irradiation Attenuates Atopic Dermatitis via Enhancing HIF-1α-Induced MANF Transcription Expression
Source: Front Immunol. 2022 Jul 14;13:941219. doi: 10.3389/fimmu.2022.941219 (PMC9329666; doi:10.3389/fimmu.2022.941219)
Supplement: Supplementary file 1 [file DataSheet_1.pdf]

Supplementary Figure 1

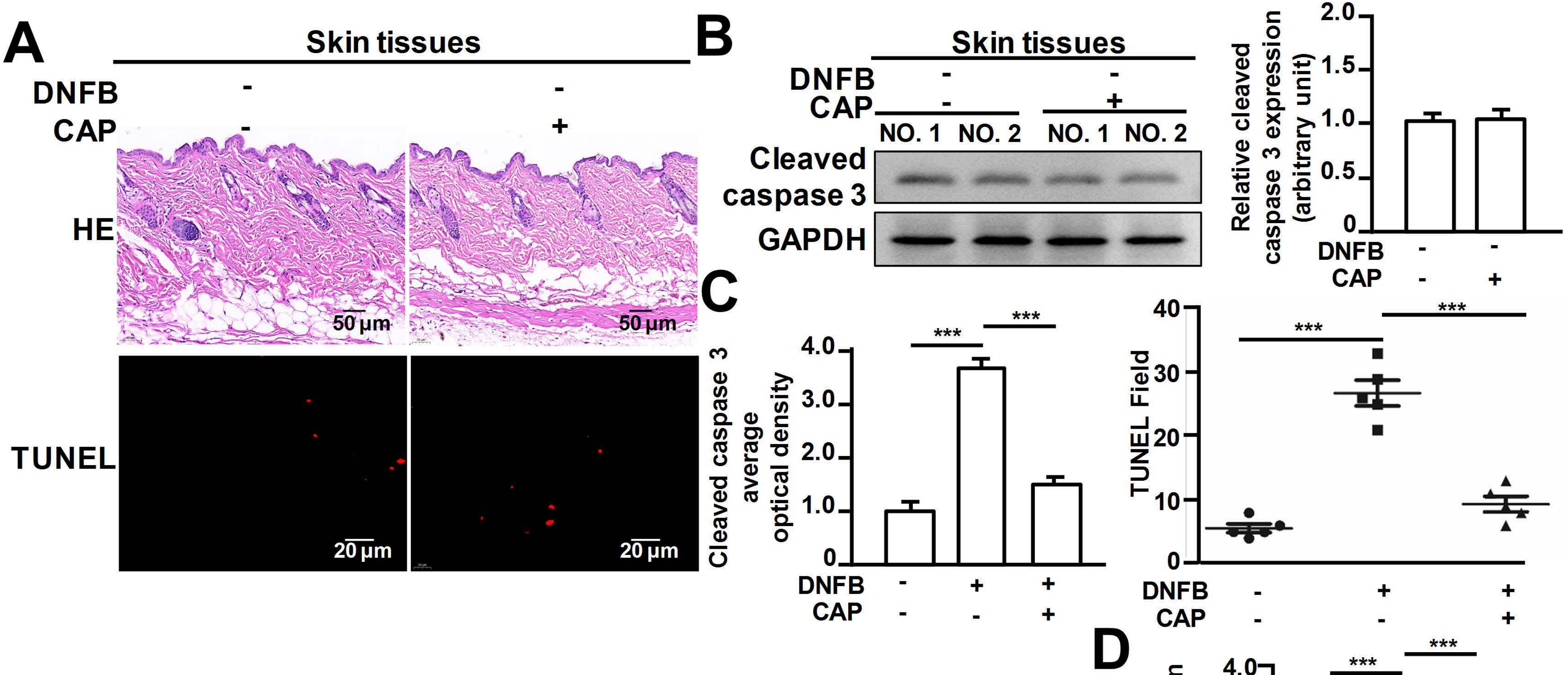

Supplementary Figure 1. CAP alleviated DNFB-induced skin injury via attenuating apoptosis. The normal mice were treated by CAP, n=8. Skin tissues (n=5) were used for HE staining, TUNEL assay (A) and western blot (B) of cleaved caspase 3. DNFB-induced AD mice model was constructed, followed by CAP treatment, n=8. Skin tissues (n=5) were used for immunohistochemical staining of cleaved caspase 3 and TUNEL assay (C), as well as western blot of cleaved caspase 3 (D). GAPDH serves as control for normalization. The average optical density, TUNEL positive field and relative protein expression were analyzed. Data are expressed as mean±SEM. *** $p<0.001$.

Supplementary Figure 2

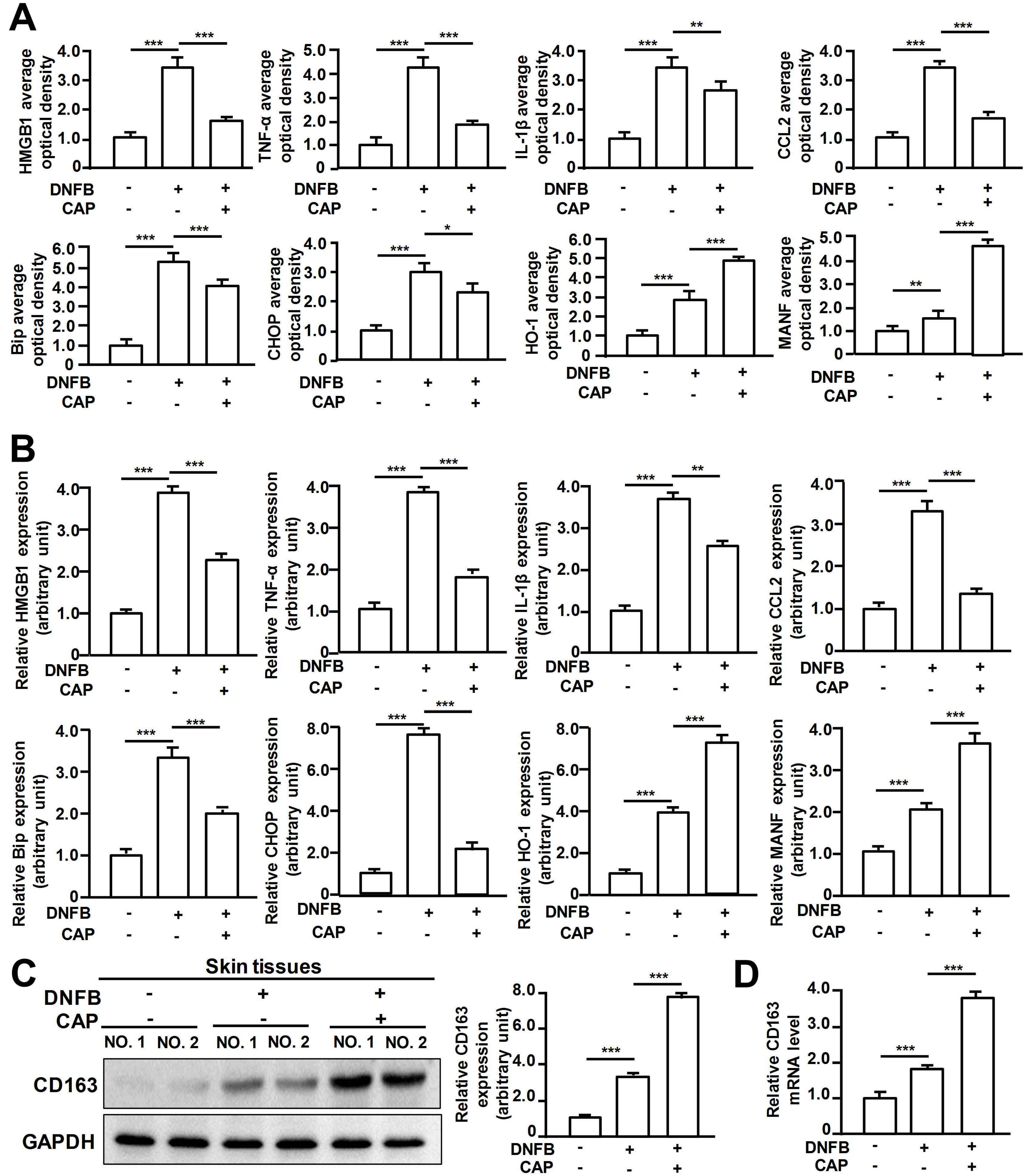

Supplementary Figure 2. CAP reduced DNFB-induced skin inflammation, ER stress, oxidative stress and proinflammatory immune cells. DNFB-induced AD mice model was constructed, followed by CAP treatment, n=8. Skin tissues (n=5) were used for immunohistochemical staining of HMGB1, TNF-α, IL-1β, CCL2, Bip, CHOP, HO-1 and MANF (A), as well as western blot (B) of the indicated proteins. Also, skin tissues (n=5) were used for western blot (C) and RT-qPCR (D) of CD163. GAPDH serves as control for normalization. The average optical density and relative protein expression were analyzed. Data are expressed as mean±SEM. * $p<0.05$, ** $p<0.01$, *** $p<0.001$.

Supplementary Figure 3

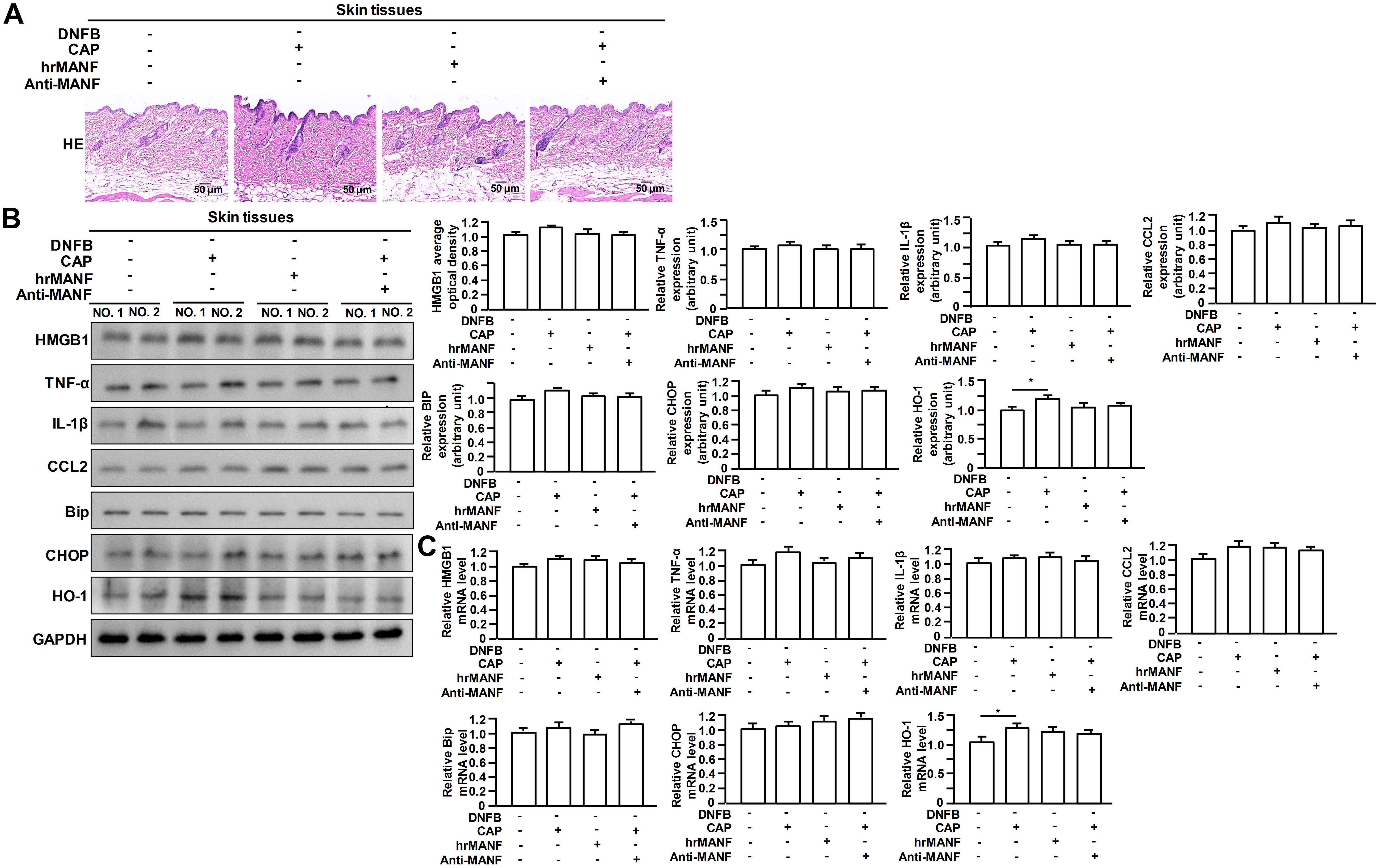

Supplementary Figure 3. CAP, hrMANF and MANF antibody treatment alone did not significantly affect skin inflammation, ER stress and oxidative stress in normal mice. The normal mice were treated by CAP, hrMANF and MANF antibody alone, n=8. Skin tissues (n=5) were used for HE staining (A), western blot (B) and RT-qPCR (C) of HMGB1, TNF-α, IL-1β, CCL2, Bip, CHOP and HO-1. GAPDH serves as control for normalization. The relative protein expression were analyzed. Data are expressed as mean±SEM. * $p<0.05$.

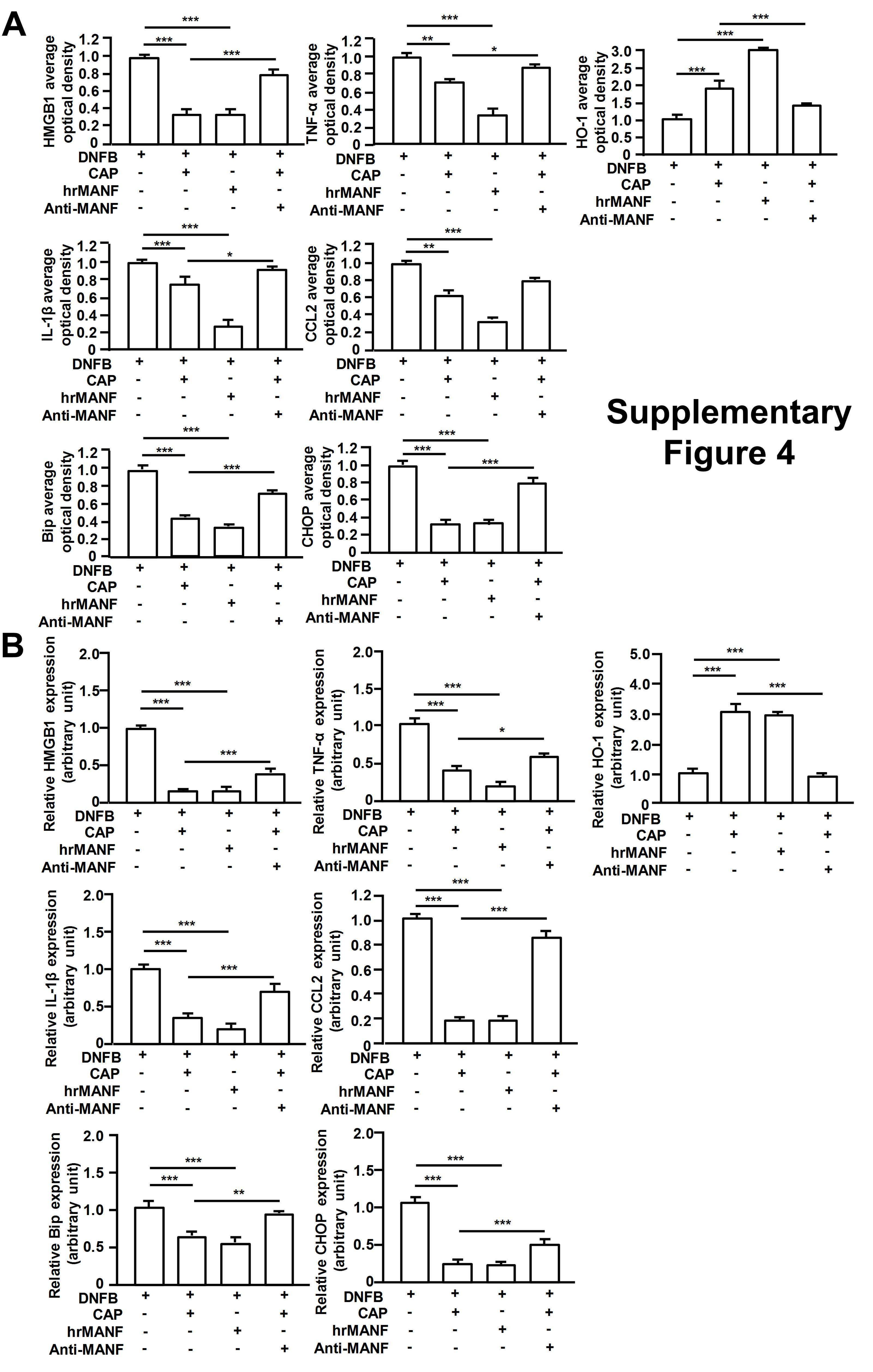

Supplementary Figure 4. CAP relieved DNFB-induced skin inflammatory injury, ER stress and oxidative stress via promoting MANF expression. DNFB-induced AD mice model was constructed, followed by CAP treatment, hrMANF treatment and MANF antibody treatment, n=8. Skin tissues (n=5) were used for immunohistochemical staining of HMGB1, TNF-α, IL-1β, CCL2, Bip, CHOP and HO-1 (A), as well as western blot (B) of the indicated proteins. GAPDH serves as control for normalization. The average optical density and relative protein expression were analyzed. Data are expressed as mean±SEM. * $p<0.05$, ** $p<0.01$, *** $p<0.001$.

Supplementary Figure 5

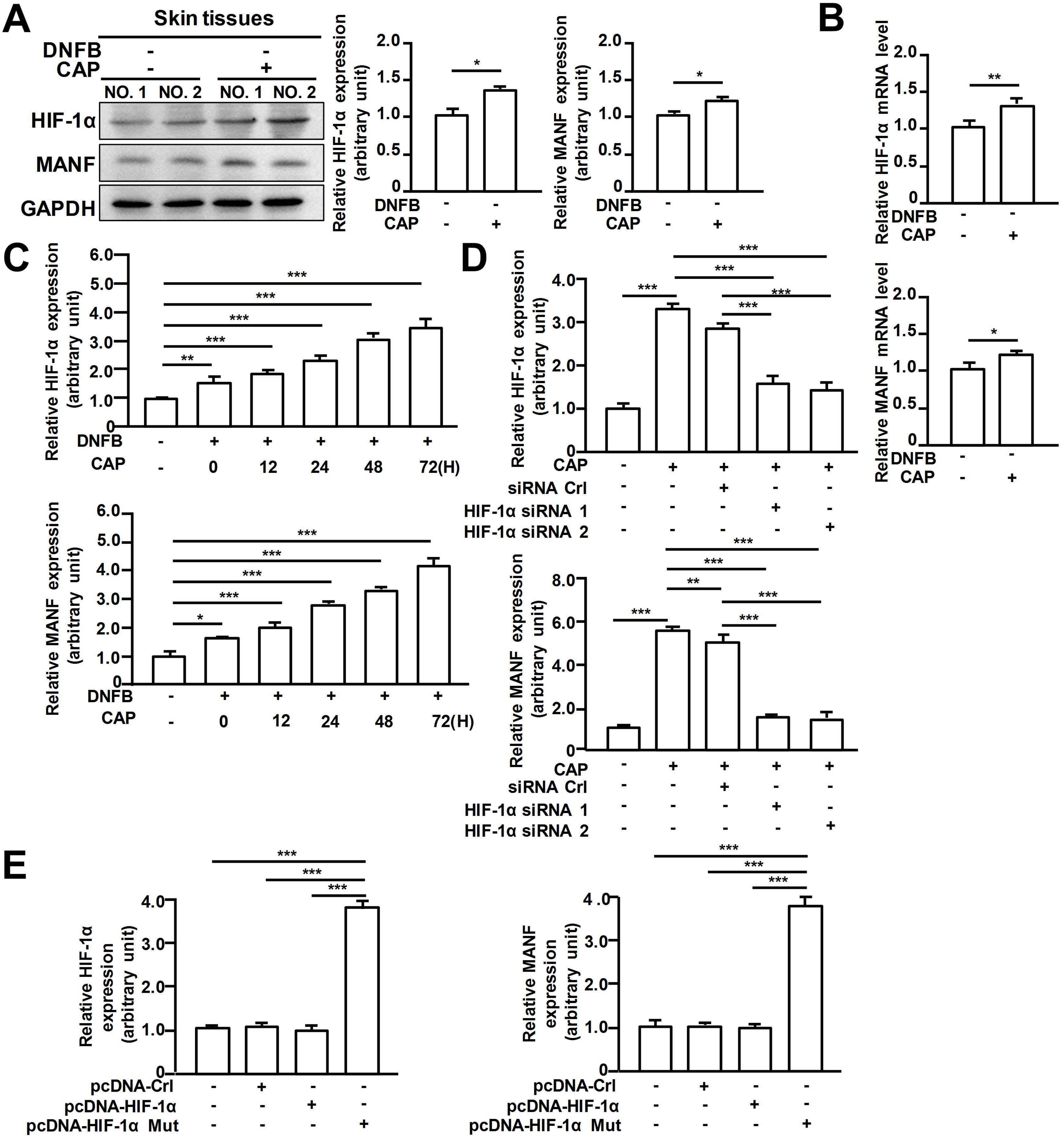

Supplementary Figure 5. CAP induced MANF transcription and expression by increasing HIF-1α level. The normal mice were treated by CAP, n=8. Skin tissues (n=5) were used for western blot (A) and RT-qPCR (B) of HIF-1α and MANF. (C) DNFB-induced AD mice model was constructed, followed by CAP treatment, n=8. At 0, 12, 24, 48 and 72 hours after CAP treatment, skin tissues (n=5) were used for western blot of HIF-1α and MANF. (D) HaCaT cells transfected by siRNA control, HIF-1α siRNA 1 and HIF-1α siRNA 2 respectively were treated by CAP, followed by western blot of HIF-1α and MANF. (E) HaCaT cells transfected by pcDNA-control, pcDNA-HIF-1α and pcDNA-HIF-1α mutation plasmid respectively were treated by CAP, followed by western blot of HIF-1α and MANF. GAPDH serves as control for normalization. The relative protein expression was analyzed. All experiments were performed independently at least three times. Data are expressed as mean±SEM. * $p<0.05$, ** $p<0.01$, *** $p<0.001$.
